# An age-related decline in the cholinergic synaptic response may cause the firing pattern in the jaw-closing motor neurons, which resembles the aversive taste response in the feeding behavior of old *Aplysia kurodai*

**DOI:** 10.1007/s00359-022-01573-y

**Published:** 2022-09-14

**Authors:** Tatsumi Nagahama, Motohiro Muramatsu, Setsuko Nagahama

**Affiliations:** 1 Department of Biophysics, Faculty of Pharmaceutical Science, Toho University, Funabashi, 274-8510 Japan; 2 Faculty of Health and Medical Science, Teikyo Heisei University, Tokyo, 170-8445 Japan

**Keywords:** *Aplysia*, Feeding, Aging, Cholinergic synapse, Functional decline

## Abstract

Anorexia due to aging is recognized as a syndrome of animal feeding behavior. Age-related functional disorders of the brain often cause behavioral changes. We used *Aplysia kurodai* to study this neural mechanism, following our previous study on food preference behaviors. The age of each wild animal was defined by a previously described method, and a significant age-related decline in food intake was observed. In this study, we explored the effects of aging on a specific inhibitory synaptic response in jaw-closing (JC) motor neurons produced by cholinergic multiaction (MA) neurons, the size of which determines the delay between MA and JC firings and this delay is reduced during aversive taste responses; in our analyses, we found a significant age-related decline in the synaptic response. Thereafter, we further explored whether such functional decline affects the JC firing pattern during the normal feeding response. During the feeding-like rhythmic responses induced by electrical nerve stimulation, the firing of the JC motor neurons advanced toward that of the MA burst, which typically happens during aversive taste responses. These results suggest that the age-related decline in the cholinergic synaptic response may partly cause the JC firing patterns that resemble the aversive taste response in old animals.

## Introduction

The aging process in animals is associated with a progressive loss of function across multiple systems. Anorexia due to aging is a syndrome of animal feeding behavior characterized by reduced food intake and body mass (Morley and Silver 1988; Chapman et al. 2002; Horwitz et al. 2002). In addition, several types of senile dementia in mammals have been reported to be responsible for changes in appetite and/or food preferences (Ikeda et al. 2002). Functional disorders of the brain with normal or abnormal aging may cause these behavioral changes. However, few studies on the changes in the activity and connections of individual neurons constituting feeding neural circuits have been performed.

The marine gastropod *Aplysia* is a well-known model for studying the neural basis of complex behaviors (Kandel 1976), mainly focusing on learning (Bailey and Kandel 2008; Lee et al. 2008; Glanzman 2009) and feeding behaviors (Nagahama and Shin 1998; Cropper et al. 2004; Baxter and Byrne 2006). This animal has a short lifespan of approximately 1 year and is a useful model for studying the neural basis of behavioral changes with aging. *A. californica* can be raised from eggs in the laboratory (Kriegstein et al. 1974). Therefore, age-dependent changes in behavior and neural functions for learning in the gill-withdrawal reflex have been well studied in this species (Lukowiak and Peretz 1980; Peretz et al. 1982; Bailey et al. 1983; Srivatsan et al. 1992; Srivatsan and Peretz 1996).

For many years, our research group has studied the neural mechanisms underlying the food preference behavior of the East Asian species *A. kurodai* and discovered significant neural pathways via several identified neurons (Nagahama and Takata 1988; Nagahama and Shin 1998; Nagahama et al. 1999; Narusuye and Nagahama 2002). We previously found that *A. kurodai* also has a lifespan of approximately 1 year (Nagahama et al. 2016). Therefore, it is of great interest to determine how neural pathways are affected by aging. *A. kurodi* cannot be raised in the laboratory and thus, the precise age of individual animals collected from the wild is unknown. Therefore, in a previous study, we devised a method to define old wild *A. kurodai* using a newly found index of old age and demonstrated that the amount of food intake significantly decreased with aging, accompanied by mass loss (Nagahama et al. 2016). These results show similar symptoms of anorexia with respect to aging. Consequently, *Aplysia* may be a useful model for elucidating this syndrome and its potential therapies. Therefore, we further investigated the neural mechanism underlying the decline in food intake in old animals.

In studies on age-related behavioral changes, synaptic dysfunction in the brain has been reported in mammals (Bartus et al. 1982; Rozycka and Liguz-Lecznar 2017; Gasiorowska et al. 2021) and *Aplysia* (Southall et al. 1997; Chandhoke et al. 2001; Kempsell and Fieber 2014, 2015). In this study, the effects of aging on synaptic function in the feeding neural circuit were explored. We then focused on a specific synaptic response in jaw-closing (JC) motor neurons produced by cholinergic buccal multiaction (MA) neurons (Nagahama and Takata 1989, 1990). We have previously demonstrated that the activity of this inhibitory synapse is reduced during the aversive taste response, and this reduction causes a change in the firing patterns in the JC motor neurons and patterned jaw movements (Nagahama and Shin 1998; Nagahama et al. 1999). The MA neuron is considered a component of the specialized neural circuit that yields the rhythmic firing patterns in the buccal motor neurons during the feeding response, generally known as a central pattern generator (CPG) (Marder and Calabrese 1996). Therefore, it is possible that the outputs from the feeding CPG are largely affected when the function of this synapse changes with aging. In this study, we initially explored the effect of aging on the function of this cholinergic synapse and then on the firing pattern in JC motor neurons during the rhythmic response induced by activating the feeding CPG directly.

## Materials and methods

### Animals

*A. kurodai* was collected from the southern coast of Miura Peninsula, Kanagawa, Japan. The animals were maintained in aquaria filled with filtered and aerated artificial seawater at a temperature of 14–16 °C. The animals were fed *Ulva* or *Undaria* once daily.

### Classification of mature and old animals

The collected animals were classified into mature and old animals using a newly found index of “old age” as reported previously (Nagahama et al. 2016). *A. kurodai* living on the coast of the Miura Peninsula can be collected from the end of November to the beginning of July every year. We commonly find a large amount of egg mass and dead bodies on the coast during and after the second half of May. Therefore, we roughly classified animals collected before and after the second half of May as mature- and old-CP animals (CP: collection period), although the mature and old animals cannot be easily discriminated by the collection period because of the distribution of the animal birthdates. In addition, we found that body mass decreased after May, but internalized shell length was maintained in each animal. The plots of the maximum shell length (*S* in mm) against the body mass (*B* in g) gave significantly distinct best-fit curves for mature-CP $(S=5.84 \times { B}^{0.354}, n=134)$ and old-CP $(S=8.22\times {B}^{0.309}, n=73)$ animals, in which the best-fit curve for old-CP animals was located above the best-fit curve for mature-CP animals. In the plots of data, the partial data for mature- and old-CP animals coexisted between two best-fit curves, but most of the remaining data for mature-CP animals existed below the best-fit curve for mature-CP animals, while most of the remaining data for old-CP animals existed above the best-fit curve for old-CP animals. In classification, the animals were considered mature when the plots of the shell length against body mass were located below the best-fit curve for mature-CP animals. In contrast, the animals were considered old when the plots were located above the best-fit curve for old-CP animals. Therefore, in this study, the internalized shell was removed from its attachment at the base of the mantle. The maximum shell length and body mass of the same animals were measured. We determined whether mature or old by comparing the measured shell length with the shell length obtained by substituting the measured body mass into the equation representing the best-fit curve.

### Preparations

The animals were anesthetized by injecting isotonic magnesium chloride solution (20–30% of body mass) into the body cavity at room temperature. The isolated preparation comprised the buccal ganglia and buccal musculature containing muscles innervated by the JC motor neurons (Nagahama and Takata 1988). The buccal mass was cut in half along the midline to separate the paired symmetrical musculature. The peripheral nerves were severe except buccal nerves 2 and 3. The recording chamber was divided into three compartments. The ganglia and paired muscle preparations were pinned to the Sylgard (Dow) surface of the separate compartments, and petroleum jelly (Vaseline) was placed on the partitions between the adjacent compartments to avoid mixing the solutions of these compartments. The sheath overlaying the buccal ganglia was surgically removed using fine scissors and forceps to expose the cell bodies of the neurons.

### Electrophysiology

The neurons were impaled with low-resistance (3–5 MΩ) glass microelectrodes filled with 2 M potassium acetate. The membrane potential of the JC motor neuron was also clamped at − 40 mV using the two-microelectrode method in voltage-clamp measurements. We previously identified 3 MA1 neurons (MA1a-MA1c) and 3 JC motor neurons (JC1-JC3) in the hemiganglion (Nagahama and Takata 1988, 1989). In the present experiments, a pair of cell bodies of MA1c neurons and JC2 motor neurons on the ipsilateral side was used to measure postsynaptic potentials and currents. We also compared the current density in voltage-clamp measurements, in which the synaptic current was divided by the membrane capacitance (Cm) in each preparation. The Cm was determined by measuring the constant current that flowed during a voltage ramp generated under voltage-clamp conditions (Neher 1971; Johnson and Thompson 1989). The artificial seawater used in the experiments had the following composition (mM): NaCl, 470; KCl, 11; CaCl_2_, 11; MgCl_2_, 25; MgSO_4_, 25; and Tris–HCl, 10 (pH 7.8–7.9).

### Analysis of the firing patterns in the neurons during the feeding-like responses

Feeding-like rhythmic patterned activities of the studied neurons were artificially elicited via repetitive electrical stimulation (duration, 5 ms; intensity, 6 V; frequency, 2 Hz) of the esophageal nerve on the ipsilateral side of the cell bodies of the studied neurons using paired wire electrodes. The typical firing patterns in the JC motor and MA neurons at the same depolarizing phase are presented in Fig. 4c, where the length of depolarization (Depo-length), delay time of the firing onset (Delay) in the JC motor neurons, and burst length (Burst-length) in the MA neurons are explained. In the analysis of the firing pattern, we also used two normalized values of time length. One value was the normalized Delay obtained by dividing the Delay by the Depo-length at each depolarizing phase (Nagahama and Shin 1998). The other value was the normalized Burst-length obtained by dividing the Burst-length by the Depo-length at each depolarizing phase at the same time in these neurons. We also analyzed the period of the rhythmic response and frequency of MA firing at each burst of the depolarizing phase.

### Statistical analysis

In this study, data are expressed as the means ± SEs. The significance of the difference between two samples was determined using a two-sample t test or Welch’s test after exploring whether distributions of two samples had homoscedasticity using an F test. Differences at *P* < 0.05 were considered significant.

## Results

### Comparison of the cholinergic synaptic responses in mature and old animals

In the present study, the effect of aging on synaptic function in the feeding neural circuit was explored. We focused on the inhibitory monosynaptic response in the JC motor neurons produced by the cholinergic MA neurons. To suppress the polysynaptic activity in these experiments, we used a solution with increased divalent cations in which Ca^2+^ and Mg^2+^ concentrations were increased, respectively, five- and twofold by substitution of Na^+^.

**Fig. 1:**
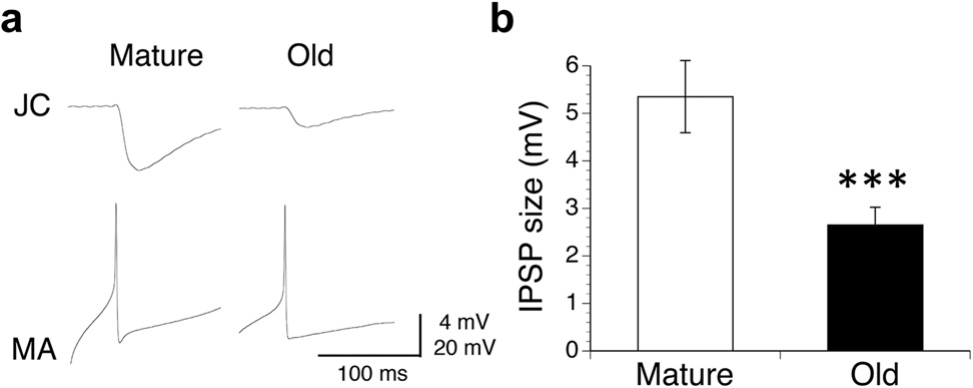
Effects of aging on the inhibitory postsynaptic potentials (IPSPs) in the JC motor neurons produced by firing of MA neurons. **a** Typical MA-produced IPSPs in JC motor neurons in mature and old animals. **b** Comparison of the average sizes of the IPSPs in the mature (*n* = 11) and old (*n* = 11) animals. ^***^ significant difference at *P* < 0.005

Figure 1a shows the typical inhibitory postsynaptic potentials (IPSPs) produced in JC motor neurons by firing of MA neurons in mature and old animals. For each preparation, the average size of the IPSPs was obtained from six trials of MA firing at 40 s intervals. This time interval was sufficient to prevent activity-dependent synaptic suppression. Figure 1b shows a comparison of the average size of the IPSPs obtained from all preparations in the mature (5.35 ± 0.76 mV, *n* = 11) and old animals (2.65 ± 0.37 mV, *n* = 11). The average size of the IPSPs was significantly smaller in the old animals than in the mature animals (*P* < 0.005). We also compared the resting membrane potentials of the JC motor neurons because the sizes of the postsynaptic potentials were affected by the membrane potentials of these neurons. In our analysis, we could not find a significant difference in the average resting membrane potential of the JC motor neurons between the mature (− 57.8 ± 1.1 mV, *n* = 18) and old animals (− 57.9 ± 1.5 mV, *n* = 18). These results indicate that the difference in IPSP size between the two age groups may be physiologically meaningful.

**Fig. 2:**
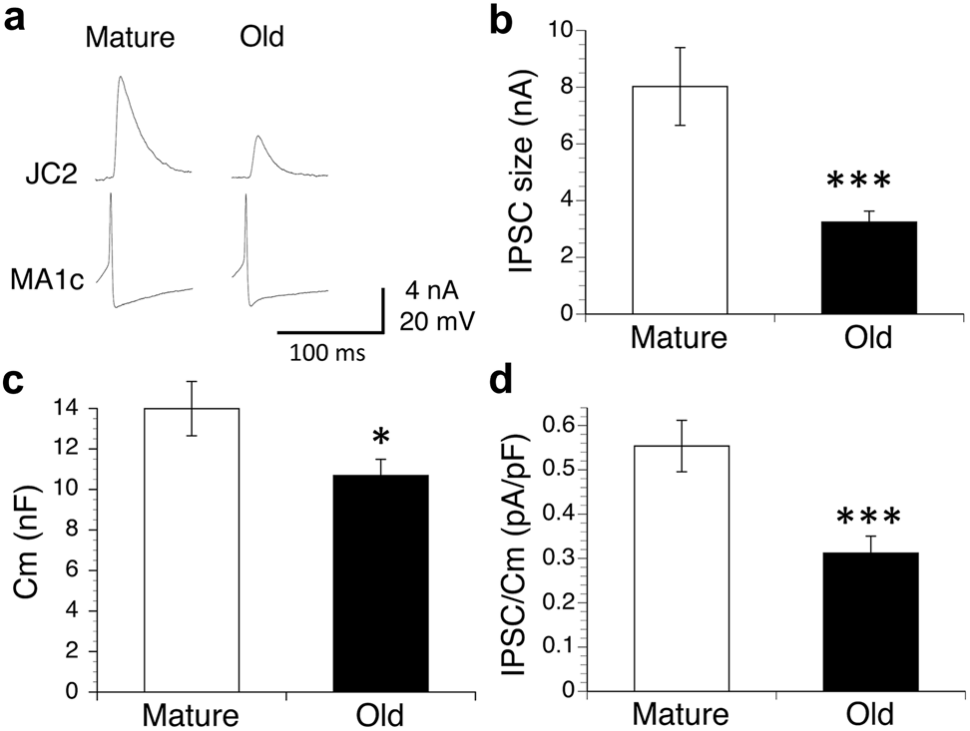
Effects of aging on the inhibitory postsynaptic currents (IPSCs) and the synaptic current density in the JC motor neurons produced by firing of MA neurons when the membrane potential of the JC motor neurons was clamped at − 40 mV in voltage-clamp measurements. **a** Typical MA-produced IPSCs in JC motor neurons in mature and old animals. **b** Comparison of the average sizes of the IPSCs in the mature (*n* = 13) and old animals (*n* = 10). **c** Comparison of the average membrane capacitance (Cm) in the JC motor neurons in the mature (*n* = 13) and old animals (*n* = 10). **d** Comparison of the average current density (IPSC/Cm) in the mature (*n* = 13) and old animals (*n* = 10). ^*^significant difference at *P* < 0.05; ^***^significant difference at *P* < 0.005

In voltage-clamp measurements, the MA-produced synaptic currents in the JC motor neurons were also compared in the mature and old animals. Then the membrane potential of the JC motor neurons was clamped at − 40 mV because the inhibitory effects of the MA neurons on the JC motor neurons during the rhythmic feeding response usually appeared near the firing threshold of the JC motor neurons (Nagahama et al. 1999). Figure 2a shows the typical inhibitory postsynaptic currents (IPSCs) produced in JC motor neurons by firing MA neurons in mature and old animals. For each preparation, the average size of the IPSCs was obtained from six trials of MA firing at 40 s intervals. Figure 2b shows a comparison of the average size of the IPSCs obtained from all preparations in the mature (8.02 ± 1.37 nA, *n* = 13) and old animals (3.24 ± 0.39 nA, *n* = 10). The average size of the IPSCs was significantly smaller in the old animals than in the mature animals (*P* < 0.005). We also explored the Cm of the same JC motor neurons, which increased in proportion to the surface area of the neurons, in the mature and old animals. As shown in Fig. 2c, the average Cm was significantly smaller (*P* < 0.05) in the old animals (10.7 ± 0.8 nF, *n* = 10) than in the mature animals (14.0 ± 1.3 nF, *n* = 13). These results suggest that the average surface area and average size of the JC motor neurons may decrease with aging. Therefore, to remove the effects of surface area on the size of the IPSC between the mature and old animals, we obtained the current density (IPSC divided by Cm) in each preparation. Figure 2d shows a comparison of the average current density in the mature and old animals. Even in this case, the average current density was significantly lower (*P* < 0.005) in the old animals (0.312 ± 0.038 pA/pF, *n* = 10) than in the mature animals (0.554 ± 0.058 pA/pF, *n* = 13). These results indicate that the cholinergic synaptic response in the JC motor neurons produced by the MA neurons may decrease with aging.

### Rhythmic firing patterns in JC motor and MA neurons induced by electrical nerve stimulation in mature and old animals

In the central nervous system of *Aplysia*, synaptic activity in other regions has also been reported to decrease with aging (Southall et al. 1997; Chandhoke et al. 2001; Kempsell and Fieber 2015). Thus, we next explored the extent to which the reduction in cholinergic synaptic activity can affect the feeding response in old animals. As reported previously, the change in this synaptic activity largely affected the rhythmic firing pattern in the JC motor neurons between the ingestive and rejective responses in food preference behavior (Nagahama et al. 1999). Therefore, we focused on the change in the rhythmic firing pattern in the JC motor neurons during the feeding response with aging. We have also previously demonstrated that electrical stimulation of any nerve leaving the buccal ganglia can directly activate the feeding CPG in isolated preparations (Nagahama and Takata 1990; Kinugawa and Nagahama 2006; Narusuye et al. 2013). Therefore, in our experiments herein, the feeding-like rhythmic firing patterns in the JC motor and MA neurons were induced via electrical stimulation of the ipsilateral esophageal nerve with repetitive short current pulses (duration, 5 ms; intensity, 6 V; frequency, 2 Hz).

**Fig. 3:**
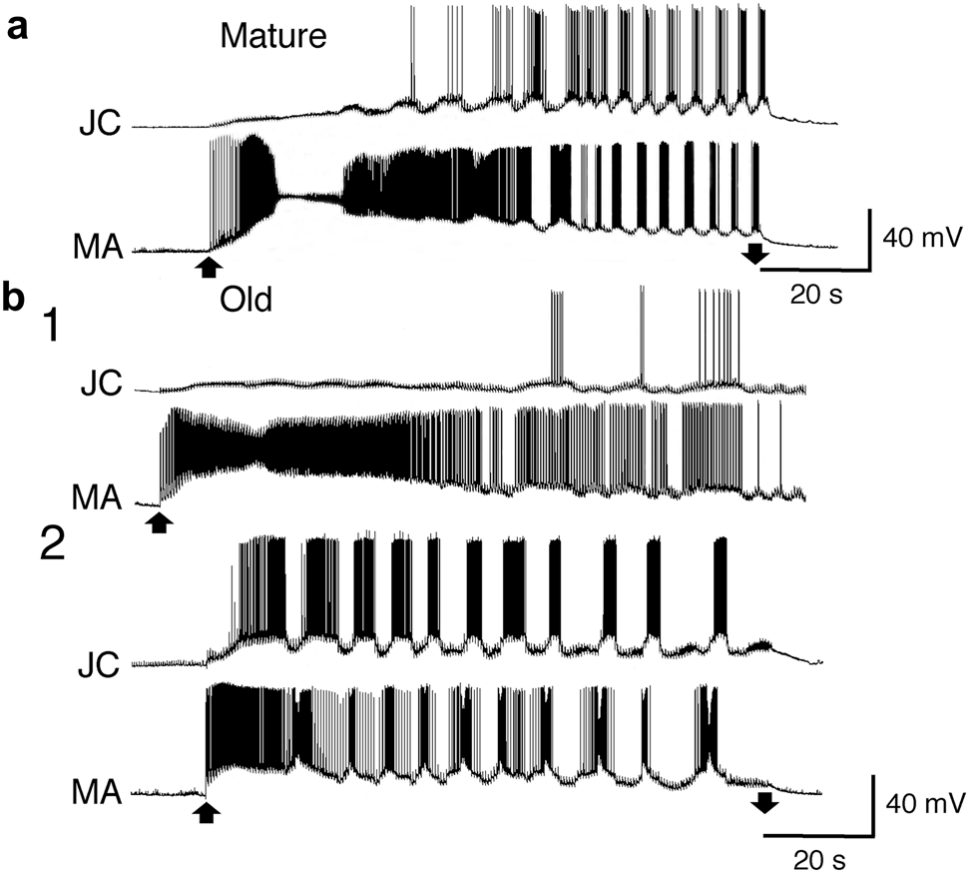
Simultaneous recordings of the rhythmic bursts of firings in the JC motor and MA neurons during the responses induced by repetitive electrical stimulation of the ipsilateral esophageal nerve in the mature (**a**) and old animals (**b2**). In the old animals, the same stimulation could not always induce stable rhythmic bursts of firing in these neurons (**b1**). Upward and downward arrows indicate the start and end of the electrical stimulation, respectively

In the mature animals, electrical stimulation induced early depolarization and successive stable rhythmic bursts of firing in both JC motor and MA neurons (Fig. 3a). However, the same stimulation did not always induce stable rhythmic bursts of firing of these neurons in the old animals, as shown in Fig. 3b1. Electrical stimulation was repeated more than 5 times in each preparation of all animals. Then, stable rhythmic responses were obtained in approximately two-thirds of all trials in each preparation of the old animals, although stable responses were always obtained in each preparation of the mature animals. Therefore, we analyzed the data representing the stable rhythmic bursts of firing in the neurons of the old animals, as shown in Fig. 3b2.

### Comparison of the periods of rhythmic responses in mature and old animals

In the analysis of the rhythmic responses, we initially explored the period, i.e., the time length between the onset times of the adjacent depolarizing phases during the rhythmic depolarization of the JC motor neurons. The period tended to elongate later in a single rhythmic response (e.g. Fig 3b2), and we obtained the value by averaging several stable successive periods for each rhythmic response. For each preparation, the average period was obtained from four trials of stimulation. Figure 5a shows a comparison of the average periods obtained from all preparations in mature (6.66 ± 0.72 s, *n* = 10) and old animals (9.67 ± 1.02 s, *n* = 13). The average period in the old animals was significantly longer than that in the mature animals (*P* < 0.02), indicating that the period was prolonged with aging.

### Comparison of firing patterns in JC motor neurons during rhythmic responses in mature and old animals

**Fig. 4:**
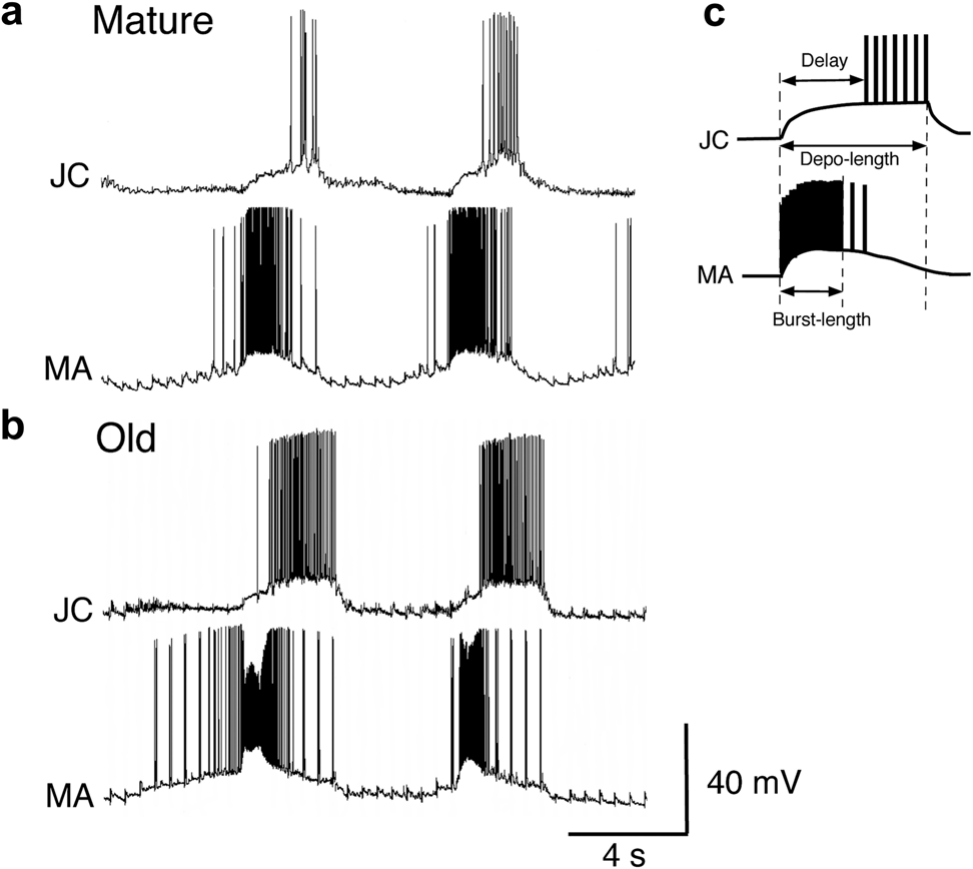
Simultaneous recordings of the typical bursts of firing in the JC motor and MA neurons during the rhythmic responses induced by repetitive electrical stimulation in the mature (**a**) and old animals (**b**). **c** Illustration of the typical firing patterns in the JC motor and MA neurons at the same depolarizing phase, explaining the length of depolarization (Depo-length), delay time of firing onset (Delay) in the JC motor neurons, and burst length (Burst-length) in the MA neurons

Simultaneous recordings of the typical bursts of firing in the JC motor and MA neurons during the rhythmic responses induced by electrical stimulation in the mature (a) and old animals (b) are shown in Fig. 4. In this figure, we found that the firing of the JC motor neurons was prominently stronger and lasted longer in old animals than in mature animals. This result can also be seen from a comparison of Fig. 3a and b2. Then, we quantitatively compared the firing patterns in the JC motor neurons between the two ages.

**Fig. 5:**
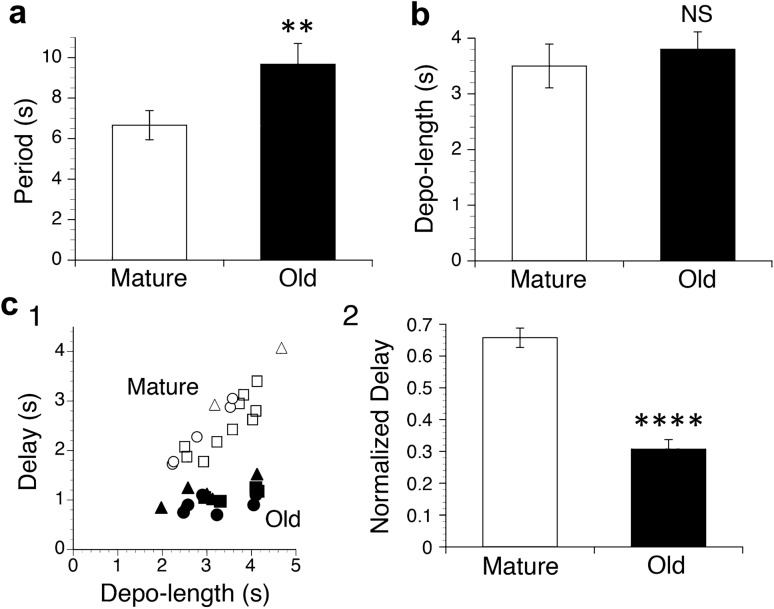
Analysis of the firing patterns in JC motor neurons during rhythmic responses in mature and old animals. **a** Comparison of the average periods of the rhythmic responses in the mature (*n* = 10) and old animals (*n* = 13). **b** Comparison of the average lengths of JC depolarization (Depo-length) in the mature (*n* = 9) and old animals (*n* = 9). **c1** Relationship between the delay time of the JC firing onset (Delay) and the length of JC depolarization (Depo-length) at each depolarizing phase during the responses induced by three trials of stimulation (different symbols) in a single mature animal (open symbols) and a single old animal (filled symbols). **c2** Comparison of the average normalized delay time of JC firing onset (normalized Delay) in mature (*n* = 9) and old animals (*n* = 9). *NS* not significant; ^**^significant difference at *P* < 0.02; ^****^significant difference at *P* < 0.001

The Depo-length at each depolarizing phase tended to fluctuate in a single rhythmic response. For each preparation, the average length was obtained from several stable successive depolarizing phases in each response and then from four trials of stimulation. Figure 5b shows a comparison of the average Depo-length obtained from all preparations in the mature (3.50 ± 0.39 s, *n* = 9) and old animals (3.80 ± 0.31 s, *n* = 9). There was no significant difference between the two age groups in the statistical analysis, although the average length was longer in the old animals.

Next, we explored the relationship between the Delay and the Depo-length at each depolarizing phase during the rhythmic responses. Figure 5c1 shows the data plots of the Delay against the Depo-length during the responses induced by three trials of stimulation (different symbols) in a single mature animal (open symbols) and a single old animal (filled symbols). These data could almost be represented by two separate straight lines, both approaching the origin for the mature and old animals, although the Depo-length fluctuated. Therefore, to analyze these data, we obtained the normalized Delay by dividing the Delay by the Depo-length at each depolarizing phase. The average value for each preparation was obtained from several stable successive depolarizing phases in each response and then from four trials of stimulation. A comparison of the average normalized Delay obtained from all preparations in the mature (0.657 ± 0.030, *n* = 9) and old animals (0.307 ± 0.030, *n* = 9) is shown in Fig. 5c2. The average normalized Delay was significantly smaller in the old animals than in the mature animals (*P* < 0.001), indicating that the onset time of JC firing at each depolarizing phase may advance with aging.

In old animals, synaptic inputs to JC motor neurons can be produced as a result of complex changes in synaptic activities and cell properties of many neurons in the neural circuit of the feeding CPG. An advance of the JC firing onset may be caused by an increase in the excitatory inputs, a decrease in the inhibitory inputs, or sometimes both, to the JC motor neurons during the early phase of JC depolarization. Therefore, the age-related decline in the inhibitory synaptic response produced by MA neurons may partly contribute to an advance of JC firing onset. However, there is a possibility that the age-related changes in some other excitatory or inhibitory inputs to the JC motor neurons could also affect the change in the firing pattern in the JC motor neurons.

### Analysis of the relationship between the firing patterns in JC motor and MA neurons during rhythmic responses in mature and old animals

The bursts of the MA firing may basically affect the firing pattern in the JC motor neurons. We next explored whether the firing pattern in the MA neurons changed with aging to affect the firing pattern in the JC motor neurons during the rhythmic responses. The Burst-length and/or firing frequency at each burst in the MA neurons at each depolarizing phase may affect the Delay in the JC motor neurons.

**Fig. 6:**
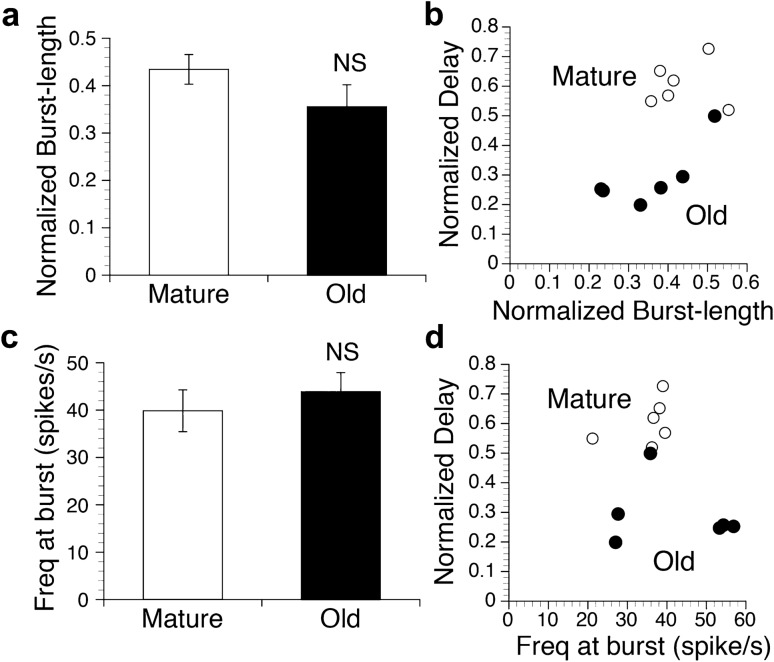
Analysis of the firing patterns in MA neurons during rhythmic responses in mature and old animals. **a** Comparison of the average normalized burst length (normalized Burst-length) in the MA neurons in the mature (*n* = 6) and old animals (*n* = 6). **b** Relationships between the normalized burst length (normalized Burst-length) and the normalized delay time of the JC firing onset (normalized Delay) for each preparation in the mature (open circles, *n* = 6) and old animals (filled circles, *n* = 6). **c** Comparison of the average firing frequency of MA neurons (Freq at burst) in mature (*n* = 6) and old animals (*n* = 6). **d** Relationships between the firing frequency of the MA neurons (Freq at burst) and the normalized delay time of the JC firing onset (normalized Delay) for each preparation in the mature (open circles, *n* = 6) and old animals (filled circles, *n* = 6). *NS* not significant

For the Burst-length, the normalized Burst-length was analyzed by dividing the Burst-length in the MA neurons by the Depo-length in the JC motor neurons at each depolarizing phase at the same time in these neurons. For each preparation, the normalized Burst-length was obtained from several stable successive phases for each response and then from four trials of stimulation. Figure 6a shows a comparison of the average normalized Burst-length obtained from all preparations in the mature (0.434 ± 0.031, *n* = 6) and old animals (0.355 ± 0.046, *n* = 6). There was no significant difference in the statistical processing between the two age groups, although the average value of the normalized Burst-length was smaller in the old animals. In contrast, the average normalized Delay in the same preparations (*n* = 6 in each age) was significantly smaller (*P* < 0.001) in the old animals (0.291 ± 0.043) than in the mature animals (0.606 ± 0.031) despite the relatively small number of preparations. To evaluate the effect of the normalized Burst-length on the decrease in the normalized Delay with aging, we also explored the relationships between the normalized Burst-length and the normalized Delay for each preparation in the same animals used for the average of the normalized Burst-length, and the results are shown in Fig. 6b. The data plots of the normalized Delay against the normalized Burst-length for each preparation showed two separate groups with large differences in the values of the normalized Delay in the mature (open circles) and old animals (filled circles). The decrease in the normalized Delay with the decrease in the normalized Burst-length in each group was not large enough to explain the large decrease in the normalized Delay in the two age groups. These results indicate that the Burst-length in MA neurons may have a little effect on the age-related decrease in the delay time of JC firing onset.

Thereafter, we explored the frequency of MA firing at each burst of the depolarizing phase using the same preparations. For each preparation, the average MA firing frequency was obtained from several stable successive phases for each response and then from four trials of stimulation. Figure 6c shows a comparison of the average MA firing frequency at each burst (Freq at burst) obtained from all preparations in the mature (39.9 ± 4.4 spikes/s, *n* = 6) and old animals (43.9 ± 4.0 spikes/s, *n* = 6). There was no significant difference in the statistical processing between the two age groups. To evaluate the effect of the MA firing frequency on the decrease in the normalized Delay in the JC motor neurons with aging, we also explored the relationships between the firing frequency and the normalized Delay for each preparation in the same animals used for the average of the MA firing frequency. As shown in Fig. 6d, the data plots of the normalized Delay against the MA firing frequency for each preparation in the mature (open circles) and old animals (filled circles) showed two separate groups with large differences in the values of the normalized Delay values. Dependence on the firing frequency of the normalized Delay value was not found in either age group. These results indicate that the MA firing frequency may have a minimal effect on the age-related decrease in the delay time of the JC firing onset.

## Discussion

### Reduction in cholinergic synaptic activity with aging

In this study, we found that the inhibitory synaptic response produced by cholinergic MA neurons in JC motor neurons significantly decreased with aging. For this synaptic transmission, we previously reported that IPSPs are blocked by d-tubocurarine, suggesting the participation of nicotinic receptors (Nagahama and Takata 1989).

In mammals, there have been many reports of significant cholinergic dysfunction in the aged brain. The cholinergic hypothesis proposed by Bartus et al. (1982) is well-known. Age-related memory loss and cognitive deficits are thought to be caused by cholinergic dysfunction. These authors also called attention to the distinction between cholinergic dysfunction during normal aging and senile dementia. During normal aging, dysfunction of the cholinergic synapses constituting muscarinic (Lippa et al. 1980; Perry 1980; Strong et al. 1980) and nicotinic receptors (Shen and Barnes 1996; Utsugisawa et al. 1999; Ghimire et al. 2020) has been reported. Our present results may be included in the results for nicotinic synapses in mammals.

In mammalian brain nuclei, many neurons have almost the same function, and only the average function of many synapses constituting pairs of the presynaptic neuron group and the follower neuron group can be explored. Conversely, individual neurons in *Aplysia* ganglia are easily identified because of the small number of neurons, and functional studies of the specific synapse constituting a pair of the identified single neurons can be easily performed (Kandel 1976). However, for the cholinergic neurons identified in *Aplysia*, few studies on age-related changes have been conducted. Moroz and Kohn (2010) explored the changes in the gene expression profiles of different single identified cholinergic neurons (R2 and LPl1) with aging and found that these two cholinergic neurons revealed highly differential genome-wide changes following aging. Their results may be helpful in understanding why some cholinergic neurons age at different rates, while others are unaffected by aging (Bartus et al. 1982). In only one study on cholinergic dysfunction, it has been reported that the excitatory response to acetylcholine and the gene expression of nicotinic acetylcholine receptors in bursting neuron R15 significantly decreased with aging (Akhmedov et al. 2013). A detailed study of age-related functional decline in the cholinergic synapses constituting a pair of identified cholinergic and follower neurons has not been performed in *Aplysia*. Our results herein will be useful for studying the mechanism of age-related decline in the cholinergic synaptic response using a pair of MA and JC motor neurons.

### Reduction in synaptic activity involving other neurotransmitters with aging

The age-related decline in synaptic activity involving neurotransmitters other than acetylcholine has also been reported in mammals (Beaudet et al. 2015; Rozycka and Liguz-Lecznar 2017; Gasiorowska et al. 2021; Griego and Galván 2021). Even in *Aplysia*, such dysfunction has been reported in synapses involving dopamine (Southall et al. 1997; Chandhoke et al. 2001), serotonin (Flinn et al. 1997; Southall et al. 1997), and glutamate (Kempsell and Fieber 2014, 2015). These results suggest that other synapses constituting the feeding neural circuit may also be affected by aging in *Aplysia*. In our study, we explored the extent to which age-related functional decline in the present cholinergic synapse affected the rhythmic firing pattern in JC motor neurons during feeding-like responses.

### Age-related changes in the rhythmic firing pattern in JC motor neurons during feeding-like responses

In studies of *Aplysia* food preference behavior, we have demonstrated that the rhythmic firing patterns in the JC motor and MA neurons were generated by the feeding CPG, and the basic patterns were modulated by the cerebral command-like neurons, namely, CBm1 for the aversive taste response (Narusuye and Nagahama 2002) and CBm3 for the favorite taste response (Narusuye et al. 2005).

Animal aging may affect the activities of the synapses in the neural circuit concerning taste and the decision to like and dislike prior to the feeding CPG. Herein, we directly activated the CPG to determine age-related changes in CPG function alone. As shown previously, electrical stimulation of any buccal nerve can activate the feeding CPG (Nagahama and Takata 1990; Kinugawa and Nagahama 2006; Narusuye et al. 2013); in our study, we used this method in the isolated preparations.

In the old animals, we found that electrical stimulation could not always induce stable rhythmic bursts of firings in the studied neurons. Even when stable rhythmic bursts were induced, the average period was significantly longer in the old animals than in the mature animals. In our experience, the period of the rhythmic response induced by electrical nerve stimulation was usually elongated with the weakening of the strength (duration, intensity or frequency) of the current pulses (data not shown). In this method, the feeding CPG may be activated by sensory inputs from the peripheral regions. Therefore, our results suggest that synaptic inputs to the CPG from the sensory neurons may decrease with aging.

In comparison of the rhythmic firing patterns in the JC motor neurons between the two ages, the normalized Delay was significantly shorter in the old animals than in the mature animals, which indicated an advance of the firing onset of the JC motor neurons during each depolarizing phase with aging. Moreover, the rhythmic firing pattern in the MA neurons was almost unchanged in the old animals compared to those in the mature animals. These results are very similar to our previous results on the changes in the firing pattern in the JC motor neurons during the aversive taste response induced by the taste of the aversive seaweed *Gelidium* compared with the favorite taste response in food preference behavior (Nagahama and Shin 1998; Nagahama et al. 1999). In this case, the cerebral command-like neurons for the aversive taste response, CBm1, decreased the inhibitory synaptic response produced by the MA neurons in the JC motor neurons and contributed to the earlier JC firing onset during the aversive taste response (Nagahama et al. 1999; Narusuye and Nagahama 2002).

In many neurons of the feeding CPG, the synaptic activities and cell properties will change with aging. Total synaptic inputs to JC motor neurons may be produced as a result of complex changes. Therefore, the present results for the firing patterns in the JC motor and MA neurons in the old animals are very interesting because it is possible that the CPG generated completely different firing patterns in the studied neurons. The earlier JC firing onset may be caused by an increase in excitatory inputs, a decrease in inhibitory inputs, or both, to the JC motor neurons during the early phase of JC depolarization. In the present study, the effects of MA neurons on the firing pattern in JC motor neurons were explored. We have previously demonstrated that the burst of MA firing suppressed JC firing during the early phase of JC depolarization in the favorite taste response because inhibition of MA firing by current-induced hyperpolarization advanced the firing onset of JC firing at each depolarizing phase (Nagahama and Takata 1990). Therefore, the age-related decline in the inhibitory synaptic response produced by the MA neurons may partly contribute to the earlier JC firing onset. However, there is a possibility that the changes in some other excitatory or inhibitory inputs to the JC motor neurons with aging may also affect the change in the firing pattern in the JC motor neurons. To study the age-related changes in the firing pattern in the JC motor neurons during CPG-induced rhythmic responses, we should further explore the age-related changes in the synaptic inputs to the JC motor neurons from other identified buccal neurons.

In previous reports on food preference behavior, we demonstrated that the firing pattern in the JC motor neurons during the aversive taste response causes the patterned jaw movements during rejection of the seaweed (Nagahama and Shin 1998). Therefore, it is possible that the patterned jaw movements during seaweed rejection are induced in old animals when the feeding CPG is normally activated, although it is assumed that the function of the jaw-closing muscle does not change with aging. In this study, we only explored the changes in the function of the feeding CPG with aging. Changes in the activities of the entire feeding system with aging must be evaluated to investigate the mechanism underlying the decline in food intake. In the future, age-related changes in muscle functions and synaptic functions in the neural circuit concerning taste and preference should be further explored.
